# Human cancer, the naked mole rat and faunal turnovers

**DOI:** 10.1002/cam4.2011

**Published:** 2019-02-21

**Authors:** Anders Bredberg, Birger Schmitz

**Affiliations:** ^1^ Department of Laboratory Medicine Lund University Lund Sweden; ^2^ Astrogeobiology Laboratory Department of Physics Lund University Lund Sweden

**Keywords:** cancer resistance, Cretaceous‐Paleogene boundary, *Heterocephalus glaber*, human cancer excess, human mutation rate, naked mole rat, Peto's paradox, rapid human evolution

## Abstract

We argue that the human evolutionary heritage with frequent adaptations through geological time to environmental change has affected a trade‐off between offspring variability and cancer resistance, and thus favored cancer‐prone individuals. We turn the attention to a factor setting the highly cancer‐resistant naked mole rat apart from most other mammals: it has remained phenotypically largely unchanged since 30‐50 million years ago. Research focusing on DNA stability mechanisms in ‘living fossil’ animals may help us find tools for cancer prevention and treatment.

1

We put to the test a hypothesis that much of human cancer is a consequence of our evolutionary heritage, with a high level of genetic variation during periods with rapid species radiation. In the trade‐off between benefit from offspring variability and deleterious effects of most new mutations, the more cancer‐prone individuals are then favored. To the best of our knowledge, this has not been discussed earlier in the medical literature, although the existence of a similar trade‐off has been suggested by for example, Fisher in 1930^1^.

The *Heterocephalus glaber* naked mole rat (NMR) has been extensively studied because of its long life span and extremely low cancer incidence.^2^, ^3^, ^4^ Interestingly, the NMR genome has features suggesting a high level of genetic stability; for example, there has been only little of rearrangement, including a low number of transposons (25%; humans have 40%).^3^ A recent long‐term study found that the mortality rate does not increase with age, and interpreted this to indicate that NMR is a nonaging mammal,^5^ supporting that NMR somatic cells are genetically highly stable. We wish to turn the attention to a factor setting NMR apart from most other mammals which, to the best of our knowledge, has not been focused upon: it is a bona fide “living fossil” phenotypically largely unchanged since 30‐50 million years ago (Ma).^3^, ^6^, ^7^, ^8^ This remarkable persistence over time may be causally linked to a peculiar lifestyle; NMR lives strictly underground excluding it from competition with other mammals, being especially relevant during periods of sudden climate change. Other species with an underground habitat are not fully sheltered from such competition because they spend some time in open air. Thus, the NMR may not have participated in the filling of open niches, which is a process associated with genetic instability; for example, there is evidence of a 10‐fold elevated DNA base substitution rate during the first 400 000 years of large‐scale mammalian diversification following the Chicxulub asteroid impact 66 Ma and Cretaceous‐Paleogene mass extinction.^9^, ^10^ In this respect, NMR differs not least with humans which originated much later, with whole genome sequencing data suggesting unanticipated and significant adaptive changes repeatedly until as late as the last couple of 1000 years.^11^, ^12^, ^13^

It is often suggested that we can learn from evolution why the modern and affluent lifestyle of humans comes with a high disease burden, and also how a fix for cancer has already been invented by other animals.^14^, ^15^, ^16^, ^17^ There has been evolution of a just‐right for each species set of energy‐demanding homeostasis mechanisms serving the individual to stay fit, and which increases in complexity with body size and life span. One cancer‐modulating homeostasis mechanism subject to natural selection may be the basal cellular mutation rate.

There has probably been selection of both promutagenic and DNA‐protective genes, in both somatic and germ cells.^18^ The mutation rate is dynamic and varies among primates,^19^ with the spectrum of mutation types having changed in humans during as short an evolutionary time as the last 20 000 years (serving as another indicator that there has recently been extensive human genetic change).^12^, ^20^ Genetic variability in somatic cells is adjustable even within a human individual.^21^ Because it is likely that the same set of DNA stability genes is operating in both somatic and germ cells,^18^, ^22^ and because cancer has its roots in mutated somatic cells, it is conceivable that selection for germ cell genetic variability will lead to more of cancer.

It should be possible to test our hypothesis by assessing the germ‐line mutation rate in NMR captivity populations^2^, ^5^ with modern methods.^20^, ^21^, ^23^, ^24^, ^25^ A finding of a relatively low mutation rate as compared with humans and other mammals would be an indication that a heavy load of recent species radiations has left a genetic scar affecting most mammals of today. Also, an investigation of other “living fossils” such as the duck‐billed platypus mammal, the coelacanth lungfish, and the crocodile and tuatara reptiles, may possibly be informative. Although data on cancer incidence in wild animals are sparse, there have been occasional case reports on tumors in some of these “living fossils”^26^, ^27^, ^28^, ^29^, ^30^ compatible with a cancer incidence as low as in NMR. Conversely, it may be informative to determine the mutation rate also in domestic animals, for which cancer seems to be a common disease and where breeding may have selected for offspring variability.^31^, ^32^

In conclusion, we argue that the human evolutionary heritage with many relatively recent species radiations and within‐species adaptations has affected a trade‐off between offspring variability and cancer resistance. If our hypothesis can be verified, research focusing on DNA stability may help us learn from cancer‐resistant animals and find tools for cancer prevention and treatment.

## CONFLICT OF INTEREST

There is no conflict of interest.
